# One-Class FMRI-Inspired EEG Model for Self-Regulation Training

**DOI:** 10.1371/journal.pone.0154968

**Published:** 2016-05-10

**Authors:** Yehudit Meir-Hasson, Jackob N. Keynan, Sivan Kinreich, Gilan Jackont, Avihay Cohen, Ilana Podlipsky-Klovatch, Talma Hendler, Nathan Intrator

**Affiliations:** 1 The Blavatnik School of Computer Science, Tel-Aviv University, Tel-Aviv, Israel; 2 The Functional Brain Imaging Unit, Tel-Aviv Sourasky Medical Center, Tel-Aviv, Israel; 3 Sagol School of Neuroscience, Tel-Aviv University, Tel-Aviv, Israel; 4 The School of Psychological Sciences, Tel-Aviv University, Tel-Aviv, Israel; 5 Sackler Faculty of Medicine, Tel-Aviv University, Tel-Aviv, Israel; Central Institute of Mental Health, GERMANY

## Abstract

Recent evidence suggests that learned self-regulation of localized brain activity in deep limbic areas such as the amygdala, may alleviate symptoms of affective disturbances. Thus far self-regulation of amygdala activity could be obtained only via fMRI guided neurofeedback, an expensive and immobile procedure. EEG on the other hand is relatively inexpensive and can be easily implemented in any location. However the clinical utility of EEG neurofeedback for affective disturbances remains limited due to low spatial resolution, which hampers the targeting of deep limbic areas such as the amygdala. We introduce an EEG prediction model of amygdala activity from a single electrode. The gold standard used for training is the fMRI-BOLD signal in the amygdala during simultaneous EEG/fMRI recording. The suggested model is based on a time/frequency representation of the EEG data with varying time-delay. Previous work has shown a strong inhomogeneity among subjects as is reflected by the models created to predict the amygdala BOLD response from EEG data. In that work, different models were constructed for different subjects. In this work, we carefully analyzed the inhomogeneity among subjects and were able to construct a single model for the majority of the subjects. We introduce a method for inhomogeneity assessment. This enables us to demonstrate a choice of subjects for which a single model could be derived. We further demonstrate the ability to modulate brain-activity in a neurofeedback setting using feedback generated by the model. We tested the effect of the neurofeedback training by showing that new subjects can learn to down-regulate the signal amplitude compared to a sham group, which received a feedback obtained by a different participant. This EEG based model can overcome substantial limitations of fMRI-NF. It can enable investigation of NF training using multiple sessions and large samples in various locations.

## 1. Introduction

A growing body of evidence shows that online feedback of particular brain activity can facilitate volitional regulation via reinforced learning; a procedure known as Neuro-Feedback (NF) [1,2]. This rapid plasticity of the human brain was first demonstrated by successfully training individuals to regulate the relative amplitude of specific EEG frequency bands such as alpha (8-12Hz) and/or theta (4-7Hz) [3]. It was further shown that learning to up-regulate theta relative to alpha could induce relaxation in post-traumatic stress disorder (PTSD) [4]. Yet the clinical benefit of such relaxation training for PTSD symptoms remains dubious [5]. One possible reason for the limited clinical effectiveness of EEG-NF for affective disturbances may be its low spatial resolution, which hampers the targeting of deep limbic areas such as the amygdala [6]. Taking advantage of the high spatial accuracy of fMRI, recent fMRI-NF studies showed that learned control over the localized BOLD activity in the amygdala corresponds with improved emotion regulation among healthy individuals [7] and may result in reduced stress [8] and depression-related symptoms [9]. However, the clinical potential of fMRI-NF is considerably limited due to the immobility and high cost of the scanning procedure [1].

Developing an approach that integrates the superior spatial resolution of fMRI with the accessibility and temporal information of EEG could therefore have substantial clinical implications for neuropsychiatric disorders [10]. From a scientific perspective, it could allow the on-going monitoring of deep brain activity in dynamic ecological set ups.

Theory driven approaches aiming to improve the spatial resolution of EEG originally attempted to construct a forward model that traces neuronal activity from both electrophysiological and hemodynamic measures [11]. However, such approaches have yet been limited by our relatively poor understanding of the way in which the distinct aspects of neural activity measured by EEG and fMRI interact [12]. Aiming to overcome the lack of prior knowledge, several data-driven approaches attempted to construct an EEG based statistical model of localized fMRI-BOLD activity. One approach takes the EEG signal at a specific frequency band [13,14,15,16,17,18]. A second approach uses the amplitude modulations of single-trial event related potentials (ERPs) [19]. Another approach applies the previous methods following decomposition into components such as principal component analysis (PCA) and independent component analysis (ICA) [20]. In these approaches, the time delay between the EEG and the BOLD signals (derived from their physiological models) was adjusted by convolving the EEG predictor with the standard hemodynamic response function (HRF) [21]. Moreover, the predictor's high temporal resolution was reduced to the fMRI temporal resolution.

More recent data-driven approaches tried to improve EEG prediction of localized fMRI-BOLD activity in a certain region, using the BOLD signal in that region as a constraint. De-Munck et al. showed that a data-driven estimation of the hemodynamic response shape of the alpha power [22] and estimation of the hemodynamic response shape for other frequency bands (each one separately) [23], could improve the correlation of the predictor with the BOLD signal. Later event-related study showed that a linear regression including all frequency bands can improve correlation to the BOLD signal, relative to that obtained by individual bands [24]. These approaches have paved the way for the idea that the EEG may provide enough information regarding fMRI BOLD changes to be exploited for neurofeedback purposes. However, what is lacking is a full representation of the electrical features that might be unique to different locations.

In our earlier work, a regression method was applied to fMRI and EEG data, acquired simultaneously to derive an individually fitted predictor of localized BOLD activity in deep brain regions, such as the amygdala and the dorso-medial pre-frontal cortex (dMPFC) [25]. The suggested framework used fMRI readouts of these regions as a trainer to the model and applied ridge regression, which was based on time/frequency representation of EEG data where each frequency band has its own time delay. The resultant model termed “EEG finger-print (EFP)” represents frequency bands and associated time delays that correlate with BOLD activity in a certain brain region. As such, the EFP allows the prediction of fMRI BOLD activity in a pre-defined region of interest (ROI) using EEG alone. However, this approach still requires prior fMRI scanning to establish a region-specific EFP for each subject (and for each subject’s session) precluding a generic widespread use at low cost.

In the current study, we therefore aim to relieve the necessity of prior fMRI by obtaining a generic EFP model (common EFP, cEFP) applicable for different sessions and different subjects. The obtained common model is then used in closed-loop training to enable a portable NF tool for self-regulation of deep brain activity.

Constructing a single cEFP model that will be valid across different sessions and different individuals is a nontrivial task; the data may originate from multiple different distributions and can be imbalanced [26]. Using the entire data set for the model construction may lead to an extensive acceptance of outliers into the model and hence may reduce the model’s accuracy [27]. A recent methodology, known as one-class classification (OCC) [28] aims to find a model that encloses all available data samples originating from the same distribution. According to the OCC methodology samples originating from the main distribution are defined as positive class, while outliers not originating from the main distribution are referred to as negative class. OCC is often used in real-time problems, when the positive class is well characterized by instances in the training data, whereas other classes (negative class or outliers) are either absent, poorly sampled or not well defined (see review in [29]). Avoiding negative samples in OCC training may improve the model’s accuracy. However, identifying those samples is a further challenge in a reality where lack of information regarding the quality of the samples complicates the extraction of positive samples for training.

To establish a common EFP model, we suggest an assessment method to find samples, which may result from the main distribution (positive samples). The method is based on hierarchical clustering algorithm [30] applied to the estimated EFP models’ coefficients. The positive samples are then used in a one-class ridge regression training to find the common EFP model coefficients. The training operates in time/frequency representation of EEG data, where each frequency band has its own delay [25]. Further to previous work [25], the fMRI-BOLD signal in the amygdala is used as a target in the training process.

Following the construction of the cEFP, we compared its accuracy to the previously developed [25] individual EFP models. Our results demonstrate that when applied on a new session or subject, the cEFP model provides better predictions of fMRI-BOLD activity in amygdala relative to individual EFPs. Implementing the cEFP in NF training using new subjects outside the fMRI scanner further demonstrates its feasibility as a neural probe for self-regulation trainings.

## 2. Materials and Methods

As stated, we aim to obtain an EEG model to predict the brain activity in a certain region as measured by the fMRI. Our previous work demonstrated the feasibility of such a model (so called EFP) fitted separately for each subject’s session by using intra-session division into training and testing sets. Thus, different models were constructed to different sessions [25].

In this study, we tried to better understand the differences between sessions, as were exhibited by the EFP models. Each EFP model is applicable to a certain session. Thus, a common model derived from a group of similar EFP models may be applicable to a group of sessions and potentially to a larger group of subjects.

This section describes the methods used to construct the common EFP model. It begins with a short description of the experimental setup, the data used to build the model and the preprocessing procedures (for more details see [25]). The common model uses a single electrode and a single common frequency band division, which is optimal for a certain group of sessions (i.e., positive samples). This selection replaces an optimal individually selection of frequency bands and optimal electrode as in [25]. An assessment method is suggested to identify this group of positive samples to be included in the one-class training. Then, the common model construction framework is introduced. The framework uses classical robust statistical methods for model selection and validation. This includes two levels of cross validation where the external cross validation uses a leave-one-out method to divide the positive sessions into training and testing sets (as a substitute to a single session division as in [25]). The last part of this section describes how to integrate the model in an EEG-NF procedure to produce cEFP-based feedback and presents the NF experimental setup.

### 2.1. Experimental Setup and Data Used for Model Construction

The common model was constructed using data recorded during an EEG-NF experiment, which is described in detail in [6]. The experiment included three EEG-NF sessions. The first session was training outside the fMRI and the other two sessions were inside the fMRI scanner. In each session, subjects were asked to relax with eyes closed for 15 minutes. Changes in their theta/alpha activity were delivered back to the subjects as a soft tune (relaxed piano tune) via headphone. The volume was adjusted every 3 sec in accordance with the changes in their theta/alpha activity. The feedback criterion was based on a scale of 10 possible values of T/A power ranging from 0.2 to 2 with 10% increase between every two sequential values. Each of these sequential increases corresponded to a specific sound intensity increasing or decreasing inversely proportional to T/A power.

In the training session the T/A feedback was calculated using real time theta (4-7Hz) and alpha (8-13Hz) signals, which were extracted from three occipital electrodes (Oz, O1, O2) [4] and averaged every 3 seconds, to calculate the ratio. In the subsequent two sessions, three individualized neuro-feedback electrodes were selected out of eight occipital electrodes (OZ, O1, O2, P3, PZ, P4, CP1, CP2) and used to extract the relevant EEG power for feedback. The chosen electrodes have the highest T/A amplitude during the training session. The sample comprised 20 subjects (7 males and 13 females) aged 25±3.5: each subject had two training sessions, except one subject who missed one session (39 sessions in total). All participants gave their written informed consent to participate in the study. The IRB committee of the Tel-Aviv Sourasky Medical Center approved the whole study.

The raw EEG data used for constructing the T/A feedback in real-time was collected online by Brain Vision RecView (Brain Products). The RecView software makes it possible to remove MR and cardio-ballistic artifacts from the EEG data in real-time using a built-in automated implementation of the average artifact subtraction method [31,32].

Preprocessing methods were applied to the EEG/fMRI data individually for each session. Preprocessing of the fMRI data using Brain-Voyager (Brain Innovation, Maastricht, The Netherlands) included removing the first six seconds to allow steady state magnetization, slice timing correction, motion correction, normalization into Talairach space, and spatial smoothing using a Gaussian kernel (3 mm, FWHM). This small Gaussian kernel was used to accommodate inter-subject differences in anatomy while minimizing blur of activation across voxels. Preprocessing of the EEG data using the EEGLAB toolbox [33] included MR gradient artifacts and cardio-ballistic artifacts removal and down sampling to 250Hz.

Later, the fMRI readout of a certain ROI and the EEG data from selected single channel (Pz) were extracted. The EEG data was converted to a time/frequency representation. Then, the frequency resolution of the converted EEG signal was reduced by averaging into 10 selected frequency bands (as a substitute for adjusting the frequency bands individually for a session). Next, the fMRI and the EEG, which have low and high temporal resolutions, respectively, were up-sampled/down-sampled to 4Hz and normalized. The determined temporal resolution of the fMRI signal and the temporal resolution of the EEG, respectively, affect the number of parameters in the model. A more detailed model, due to higher temporal resolution, may reveal brain processes [25]. However, it increases the running-time complexity and overfitting problems. We used 4Hz to balance this trade-off. For more information on the preprocessing methods and the up-sampling effect, see [25].

After preprocessing, each time-point in the BOLD signal corresponded to a time-window in the EEG. The data representation for the model using a single channel CH is a multi-dimensional matrix [FQ]x[DELAY]x[TIME]. An activity detected by the fMRI (i.e. the BOLD response) at time *T* can be predicted in the EEG using the frequency intensity *FQ* of channel *CH* in delay *D* from *T*.

### 2.2. One-Class Modeling

This study proposes a model that represents a behavior that can be identified in the majority of the sessions. Hence, the model training should be based on positive samples having this behavior. Here, the positive samples are sessions that have similar EFPs.

We define a metric to measure the similarity between two EFPs and a transformation to bring the EFPs into one space before calculating the metric. Then, a clustering algorithm is applied on the EFPs, based on the defined metric, to extract set of similar sessions from the clustering-tree.

Two EFPs were considered more similar when the correlation between their coefficients increased. Such definition is valid in case of regression model, but not valid when permutation of the parameters of the model is possible (e.g. feed-forward neural network [34]).

**Fig 1:**
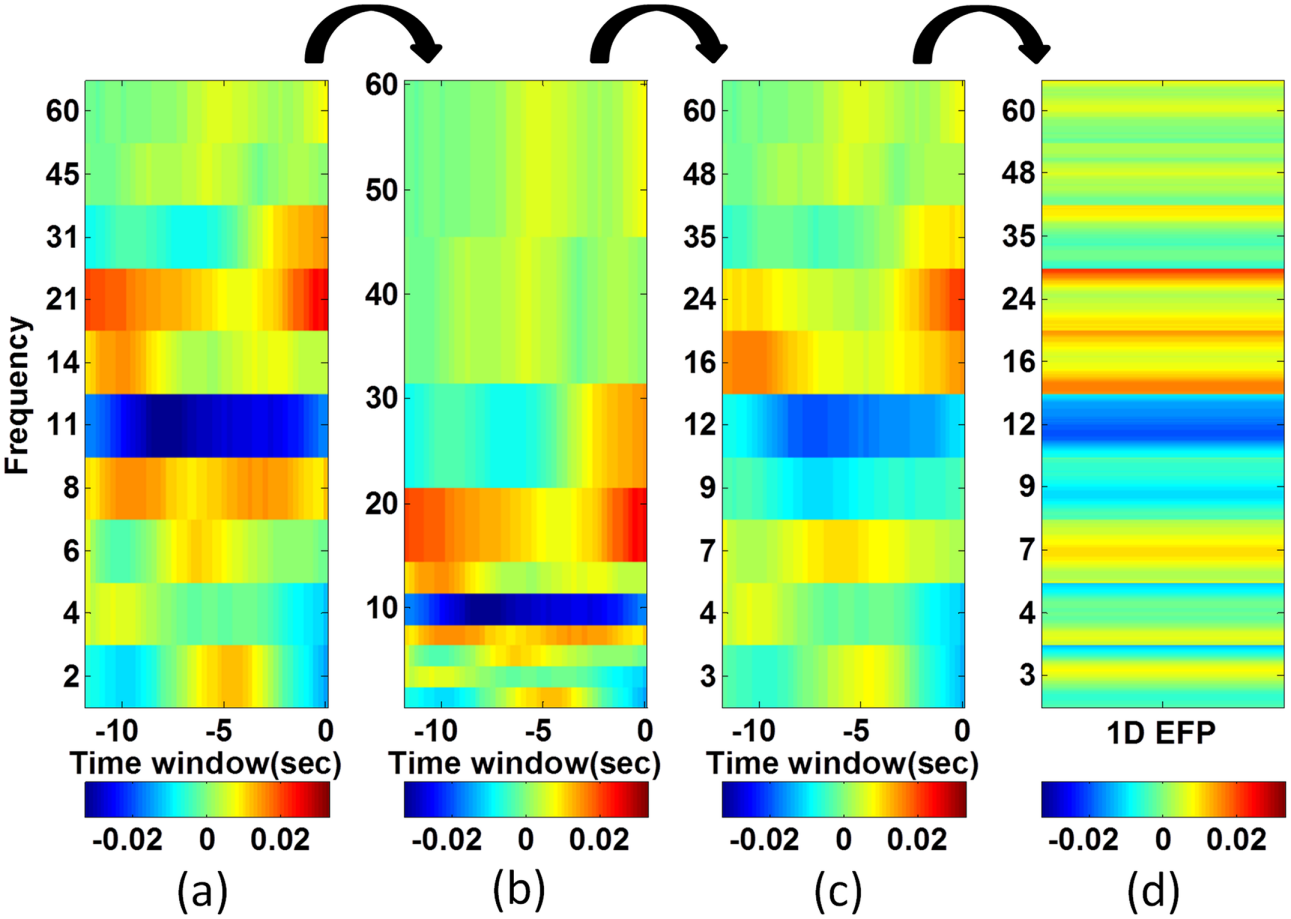
The transformation steps before applying the metric. a) The original EFP. b) Expanding y-axes to a minimum resolution of 1Hz. c) Collapsing y-axes to a uniform frequency band division. d) Reshaping EFP to a vector.

The transformation steps before applying the metric are shown in Fig 1. These include: converting their frequency bands on the y-axis to a single frequency band division by expanding their y-axes to a minimum resolution of 1Hz and collapsing this back to a uniform frequency band division. The uniform frequency bands used for comparing two EFPs divided into 10 equal areas the averaged spectral logarithmic mean of the EEG data across all sessions (instead of a single session as in [25]). Then, the resultant EFP matrix coefficients were converted to a vector of size m*n, where *m* and *n* are the width and the height of the matrix, respectively.

An iterative hierarchical clustering algorithm [30] was applied to the vectors to find EFPs with the highest similarity. The algorithm started with the vectors as separate leaves/clusters in the clustering tree. At each iteration, the clustering algorithm linked two vectors where the distance between the vectors was minimal. The distance between two vectors was defined as 1-R, where R is Pearson's correlation coefficient between the two vectors. The merged cluster was represented by a vector resulted from the averaging of the vectors in the cluster.

The clustering process was terminated when the number of clusters was 24 and observed a small knee in the objective function curve [35]. The closest n (n = 10) sessions (i.e. those with the smallest maximum internal distance between sessions), belonging to the biggest cluster, were used in the one-class training.

### 2.3. Common Model Construction Framework

The prediction framework included creating a family of models having different model constraints (i.e., regularization parameter). Each member of the family (i.e., a model with a regularization parameter) attempted to simulate the brain activity measured by the fMRI in a certain brain region by finding the model coefficients that best describe the activity in this ROI. The ‘best’ model, which best predicted the ROI activity, was selected via cross validation.

Cross-validation is a standard procedure for model selection and validation when the data is limited. The use of regularization and cross-validation may reduce overfitting, which might be caused due to the model’s increased number of parameters (Occam's razor principle). Regularization reduces overfitting by keeping feature weights relatively small. In cross-validation, the data is divided into several disjoint training and testing sets. This is used to avoid overfitting resulted from the large number of free parameters relative to the size of the training data [36].

**Fig 2:**
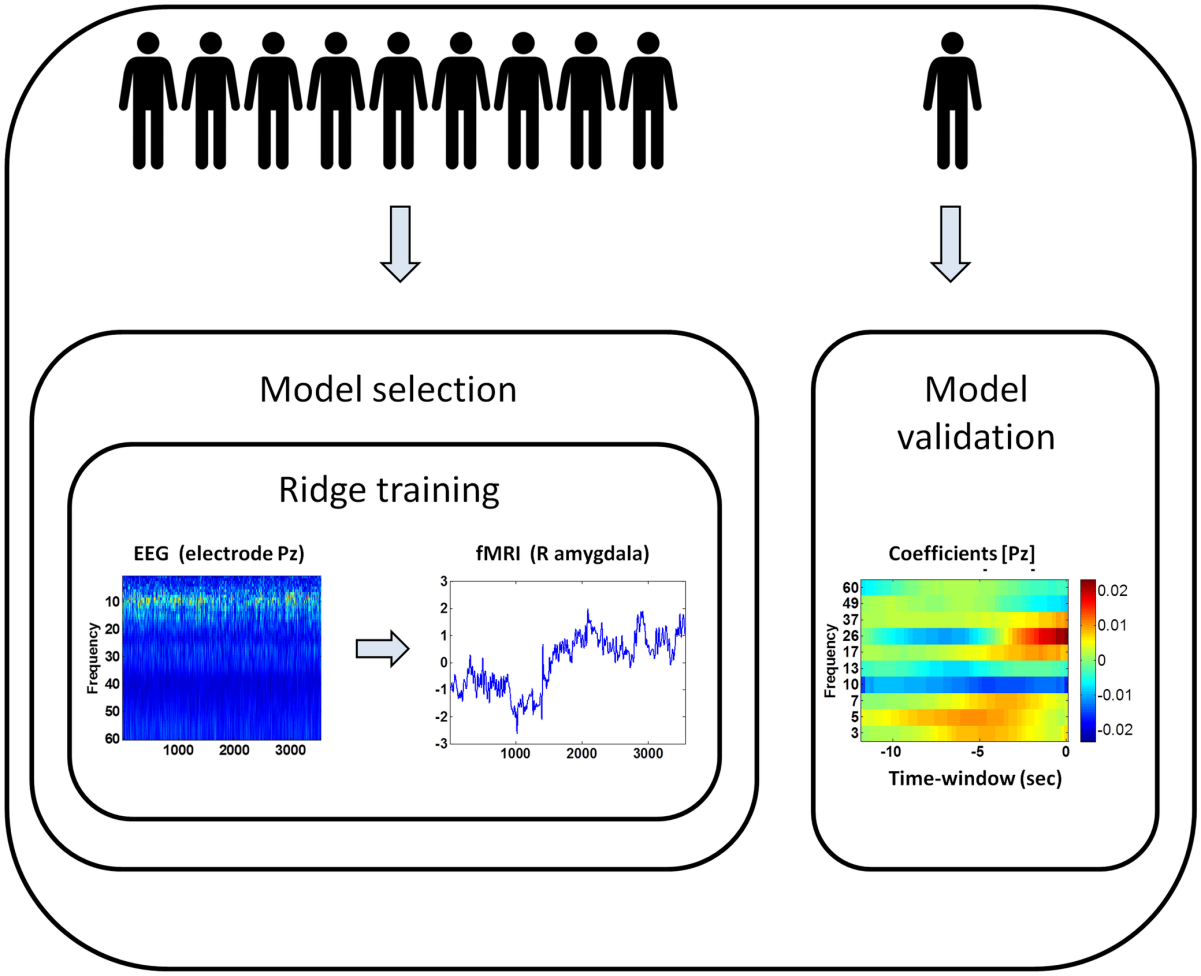
Scheme of the common model construction framework. The samples were divided in a leave-one-out manner into training and testing sets. The training set was used for model selection and the testing set was used for model validation. An inner cross-validation was used for choosing the optimal model (i.e. finding the model coefficients and the best regularization parameter) based on regularized ridge-regression training. The training input was the time-frequency representation of the EEG data and the training target was the fMRI BOLD signal in the amygdala. Each time-point in the BOLD signal corresponded to a time-window in the EEG. The resultant model coefficients suggest frequency bands and time delays that correlate to the BOLD activity in the amygdala.

In this study, two methods of cross-validation were employed (as seen in Fig 2). An external cross-validation method was used for dividing the data into training and testing sets. The training set was used for finding the optimal model and the testing set was used for checking its accuracy. An inner cross-validation method was used for selecting the optimal model (i.e., finding the best regularization parameter) on the training set.

In the external cross-validation method, the positive samples (*k* = 10, sessions belonging to different subjects) were divided in a leave-one-out manner into training and testing sets (replacing division of a single session as in [25]). *k-1* samples were considered as ‘training set’ (as a whole unit) and were used for finding the optimal model. One sample was considered a ‘test set’ and was used for checking the model’s accuracy.

In the inner cross-validation method, the training set was randomly split (in block design), *n* times (*n* = 30), into 80–20% inner-training and validation sets, respectively. The regressor ran on the inner-training set with different values of regularization parameter (within range of interest [25]). It attempted to find the model coefficients that best describe the activity measured by the fMRI in a region of interest. The model that yielded the best results on the validation set (i.e., brought the normalized mean square error to a minimum) was selected.

Model construction was based on regularized ridge-regression (RR). This linear regression method provides direct interpretation of the model: the coefficients suggest frequency bands and time delays that correlate to the ROI activity (i.e., the finger-print). Further details and comparison with other methods can be found in [25].

### 2.4. Integrating the Common EFP Model in NF Training

The ability of the model to provide an effective neural feedback for self-regulation of new subject was tested by demonstrating that participants can modulate their brain activity using feedback generated by the model.

To enable volitional regulation training of the cEFP signal, we developed custom software that records momentary changes in the signal amplitude and, accordingly, changes the audio feedback volume provided to the subject. The custom software scheme has three parts: acquiring the EEG data in real-time, using the data received to generate the next point on the cEFP signal, and returning of audio feedback to the subject corresponds to the cEFP amplitude change.

EEG data was acquired outside of the MRI scanner using the BrainAmp-MR EEG amplifier (Brain Products, Munich Germany) and the BrainCap electrode cap with sintered Ag/AgCI ring electrodes providing 30 EEG channels, 1 ECG channel, and 1 EOG channel (Falk Minow Services, Herrsching-Breitburnn, Germany). The electrodes were positioned according to the 10/20 system. The reference electrode was between Fz and Cz. Raw EEG was sampled at 5 kHz and recorded using Brain Vision Recorder software (Brain Products).

**Fig 3:**
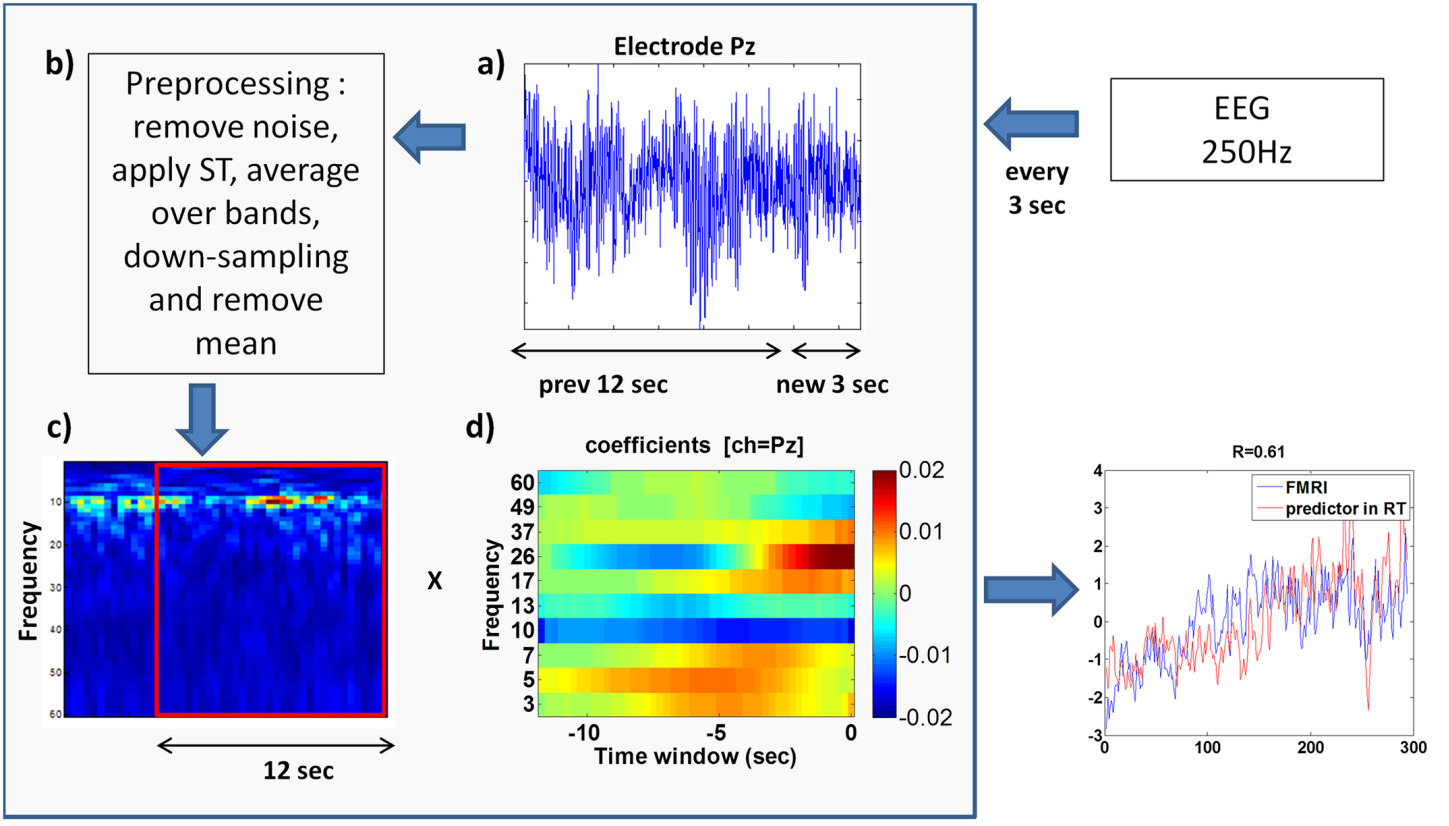
Scheme of the virtual machine used during EEG-NF training. The virtual machine receives the last 3 seconds in the EEG data and returns the predicted BOLD value that corresponds to the last change. a) New EEG segment is attached. b) Preprocessing of the buffer. c-d) Last 12 seconds are multiplied by the cEFP matrix coefficients to calculate the next point on the cEFP signal.

The virtual machine used to construct the cEFP signal during the real-time NF process is illustrated in Fig 3. The virtual machine received the last 3 seconds in the EEG data and returned the predicted BOLD value that corresponded to the last change. The specific time segment used for the cEFP model (3 seconds) was chosen to match the fMRI time resolution (TR = 3000ms). An equal time resolution between the EEG predictor and the fMRI will enable future validation of the predictive power of the cEFP model. To calculate the next cEFP value, a buffer of the last EEG time series at electrode Pz (12-second-long) was kept in the memory. When a new packet of EEG data arrived (3-second-long), it was attached to the stored buffer. Preprocessing methods applied to the united buffer in real-time were similar to preprocessing methods applied off-line to the EEG data to construct the common model. These include filtering power line noise using a notch filter at 50Hz, converting the EEG time series into a time/frequency representation using the Stockwell transformation (ST), down-sampling the transformation product to 4Hz, reducing the frequency resolution by splitting into 10 frequency bands (defined by the common EFP model), and normalizing by subtracting the mean calculated during a rest session (see [25] for additional information). After applying preprocessing methods on the buffer, the last 12 seconds were multiplied by the cEFP’s matrix coefficients to calculate the next point on the cEFP signal.

The audio feedback returned to the subject during NF period corresponded to the cEFP signal amplitude changes. The auditory feedback consisted of a 3 second-long piano musical tone. The loudness ranged from 10dB to 90dB. Volume changes were set in a linear scale, according to the real-time calculation of the cEFP signal. Before the NF periods, the participant had a rest period, which was used to calculate the participant’s mean cEFP value during rest and the standard deviation (std) across this mean. A loudness of 50dB was set during NF periods when a cEFP value equal to the mean of the rest was recorded. A change of one std in cEFP value (either up or down) caused a respective change of 10dB in the loudness of the auditory feedback. After each NF period the std was reset in accordance to the cEFP values recorded during the last NF period.

The NF experiment included thirteen healthy participants (7 males and 6 females) aged between 23 and 28 years (mean = 25.12, sd = 1.45). All participants gave their written informed consent to participate in the study. The IRB committee of the Tel-Aviv Sourasky Medical Center approved the whole study. The participants were randomly assigned to either a test (*n* = 7) or a sham (*n* = 6) group in a single blind manner.

The NF experiment comprised five periods, each lasting seven minutes. The first was a baseline (BL) period during which participants were instructed to rest with their eyes closed and received no auditory feedback. During the subsequent four NF periods, participants received continuous auditory feedback via stereo insulated headphones and were generally instructed to explore a mental exercise to lower the sound volume. Instructions were intentionally general, allowing individuals to endorse the mental exercise they found subjectively to be the most efficient [37].

For subjects in the test group, the volume of the auditory feedback was driven by their cEFP signal amplitude changes, which was calculated online every 3 seconds using the software described. The cEFP amplitude changes (up\down) were reflected by corresponding changes in sound volume (up\down). The sham group received feedback that to avoid frustration, was based on the cEFP signal modulation obtained from a different, randomly chosen participant (from a pool of 5 participants) who overall exhibited successful down-regulation. Thus, the sham group had experienced the expected modulation in the sound volume, although it was unrelated to their own cEFP signal change.

After completing the NF session, participants were asked to briefly describe the mental strategies they found to be effective in down-regulating the auditory volume. Overall, the debriefing suggested three main types of mental strategies used by the participants. Guided imagery and self-introspection were reported by the majority of subjects (8 out of 13) to be the most effective mental strategies. Imagined manipulation on the auditory stimulus (e.g., imagining it slowing down or its volume weakening) was also reported to be helpful. Importantly, none of the sham group participants suspected that they were assigned to a control condition. Furthermore, participants of the sham group reported similar mental strategies and felt they were successful.

## 3. Results

Further to previous work [25], we applied the suggested framework to the right amygdala (Talaraich [20,–5,–17], MNI [20,–4,–21], and a Gaussian sphere radius of 6 mm). Amygdala activity was detected using simultaneous recordings of EEG/fMRI, where participants were instructed to relax while receiving auditory feedback and guided online by their theta/alpha ratio modulation (see [6] and method section for additional details). The EEG-NF protocol, which was aimed at increasing the theta/alpha power ratio (T/A NF), has been used to enhance a state of deep relaxation, in a range of clinical conditions, such as post traumatic stress disorder (PTSD) [38].

### 3.1. The Amygdala’s Common Model

In our previous work, we found great variability among subjects/sessions in the electrode that provided the best prediction in terms of optimal frequency choice and modeling [25]. Given this diversity, the suggested common model does not intend to predict the BOLD activity of each subject, but to represent a common behavior reflected in the majority of sessions.

**Fig 4:**
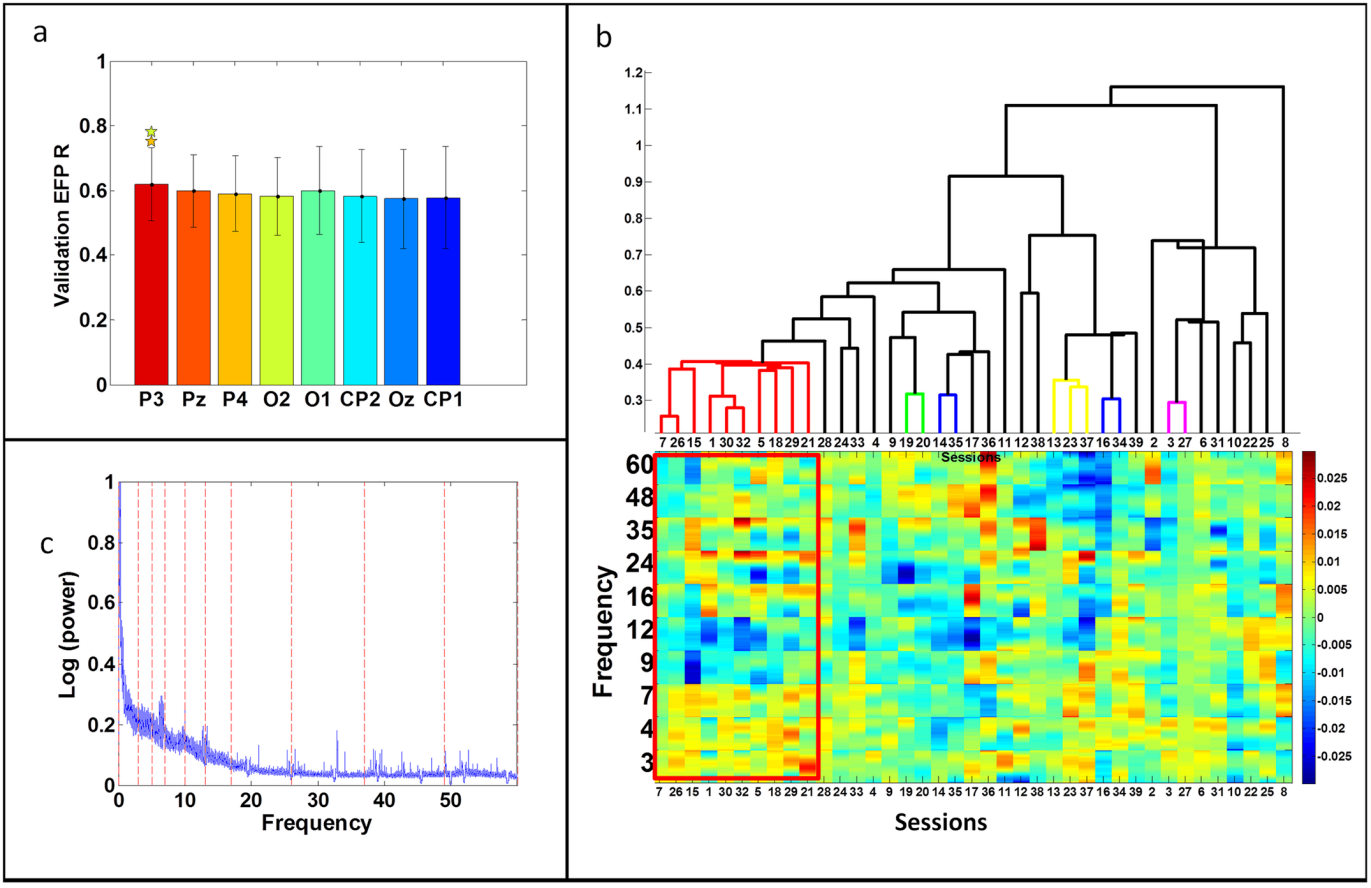
Common EFP characteristics. a) Individual EFP prediction correlation coefficient on the validation set using different electrodes on the back of the brain, averaged over all 'successful' sessions (i.e., those with prediction correlation coefficients greater than 0.6 on the validation set using any electrode, *n* = 26). The electrodes are sorted according to their signal-to-noise ratio (*μ∕σ*). b) The dendrogram of the clustering results and the EFPs’ coefficients in the leaves. The different clusters are marked in different colors. The 10 selected sessions, belonging to the biggest cluster, are marked in red. c) The cEFP frequency bands divide the averaged spectral logarithmic mean of the EEG data across the 10 selected sessions to 10 equal areas.

The common EFP for the amygdala was based on electrode Pz. Fig 4a shows the performance of different electrodes using an individual model for this task. While electrode P3 appears slightly better than the others, adjacent electrodes in more posterior regions, achieved roughly similar results (i.e., an insignificant difference at *p<0.05). The chosen electrode was, nevertheless, Pz, which is adjacent to electrode P3. Therefore, both are less sensitive to eye movements and to the "Berger effect" [39]. However, unlike electrode P3, electrode Pz is closer to the medial temporal cortex. Due to its medial location, it might be more sensitive in detecting amygdala activity in both hemispheres. In addition, recent papers dealing with T/A training using a single electrode have chosen Pz as their NF electrode [40,41].

The sessions used in the model construction had the greatest similarity between their estimated models using electrode Pz. These sessions (i.e. positive sessions) were identified using an assessment method (introduced in subsection 2.2). This method determined a metric between two EFPs and used clustering algorithm [30] to construct a hierarchical tree of the EFP models. Fig 4b depicts the dendrogram of the clustering results and the EFPs’ coefficients in the leaves. The different clusters are marked in different colors. The 10 sessions, belonging to the biggest cluster, are marked in red. These sessions, belonging to different subjects, were included in model construction.

The common EFP frequency band division was determined based on data collected from these positive sessions. The chosen frequency bands divided into 10 equal areas, the averaged spectral logarithmic mean of EEG data across the 10 selected sessions (instead of a single session as in [25], or the entire sessions as above is used for comparing individual EFPs) (Fig 4c).

The common EFP obtained for the amygdala using the suggested framework and the model characteristics described above is depicted in Fig 3d.

The performance of the common EFP was compared with the individual EFP [25] performance. The EFP was constructed using intra-session division to training and testing sets. Therefore, theoretically, it may optimally describe brain activity in that session. Since our aim is to eliminate the fMRI, two questions are raised: can an EFP created for a subject during one session predict the brain activity of the same subject during another session. Moreover, is it possible to apply an EFP created for a subject to another subject?

**Fig 5:**
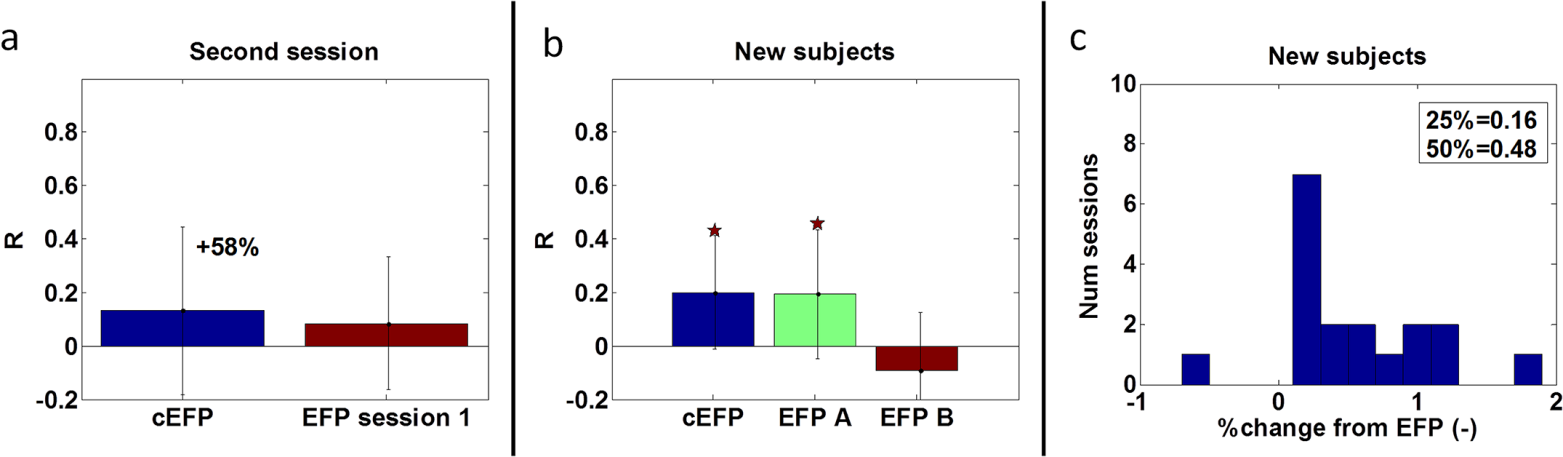
Comparison between the cEFP and the EFP performances. a) Depicts the performance of the individual model constructed for the first session when testing on the second session. It compared with the cEFP performance on the same sessions. The results are an average over subjects whose first session was included in the common model construction process (*n* = 9). b) Compares the cEFP performance with the performance of two ‘optimal’ EFPs, when applied to a group of new subjects (*n* = 18, 9 subjects). In Fig 5a and 5b, the star's color represents the method that obtained significance (*p < 0.05). The error bars are standard deviations over sessions. c) Depicts the cEFP percentage change histogram (relative to EFP).

Two sessions of the same subject recorded within a single scanner run will probably be more related (e.g. similar physical position and head motion). This lower intra-subject variability shown in [42] may enable applying a model trained on one session to another session. However, differences between sessions may have arisen from changes in the subjects' condition (fatigue, motivation), particularly in task-oriented experiments. Therefore, this adaptation cannot be automatically performed. Fig 5a depicts the performance of the individual model constructed for the first session when testing on the second session. The figure focuses on subjects for whom their first session was included in the common model construction process (n = 9). The EFP performance was compared with the cEFP performance on the same sessions. The comparison shows the superiority of the cEFP over an EFP, constructed using a previous session of the same subject.

In Fig 5b, the cEFP performance was compared with the performance of two EFPs, when applying to new subjects (n = 18, 9 subjects). Recent studies showed that the intra-subject variability [42] is lower than inter-subject variability [43]. Thus, applying a model trained on one subject to another subject may not be applicable. Therefore, the EFP models compared with the cEFP were not arbitrarily selected, but their selection was based on a record of success (in terms of prediction). The chosen EFPs, achieved in [25] the highest prediction result (‘optimal’ sessions); the session of the first (EFP A) was included in the cEFP construction and the session of the second (EFP B) was excluded. New subjects are subjects whose sessions were neither ‘optimal’ nor included in the cEFP construction. Therefore, by definition, the distance between the EFPs of the new subjects to the cEFP was greater. Results show that high EFP performance does not indicate robustness when applied to new subjects. Fig 5c quantifies the percentage change of the common model compared with the individual models. It shows that among the new subject group, 25% of the sessions had a percentage change below 0.16 and 50% of the sessions had a percentage change below 0.48. In Fig 5c, the cEFP prediction result is an average over the individual’s train-test division for a fair comparison with the individual’s score.

### 3.2. Down-regulation of the cEFP-NF signal

This section describes the performance of new subjects when down-regulating the amplitude of the NF signal, which was generated in real-time using the cEFP model. In the NF experiment, participants were instructed to relax while receiving auditory feedback and guided online by their cEFP signal amplitude changes. The test group of subjects was compared with a sham group of subjects, which received feedback based on the cEFP signal modulation obtained from different participant who overall exhibited successful down-regulation.

**Fig 6:**
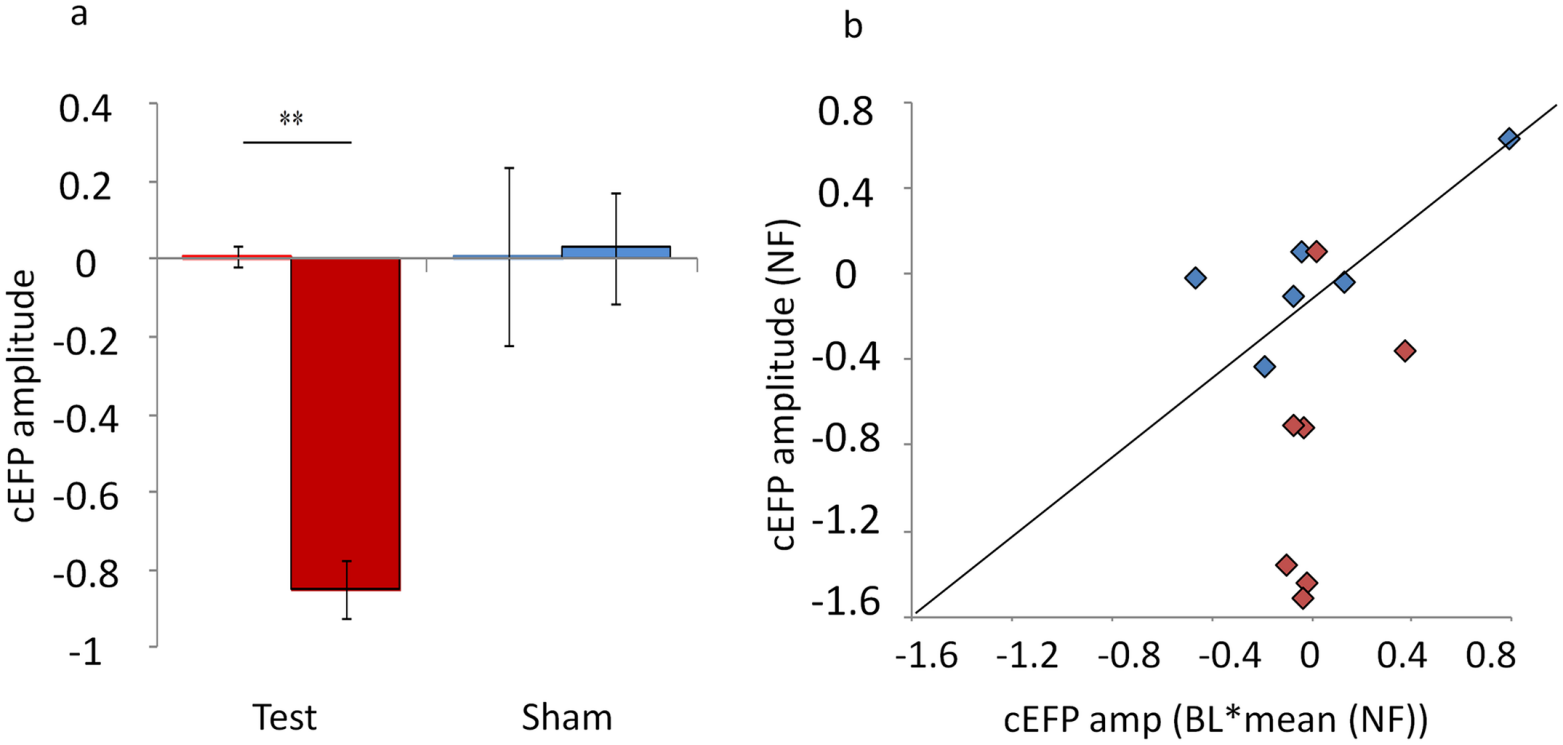
Down-regulating the common EFP signal amplitude. a) Mean results of the amygdala common EFP-NF. The *y* axis shows the mean cEFP amplitude during BL (left columns) and NF (right columns). Only the test group (red columns, *n* = 7) had significantly reduced cEFP amplitude during NF relative to BL (F(1,11) = 24.46, **p<0.01). b) Individual results of the common EFP-NF. The *y* axis shows the cEFP amplitude during NF and the *x* axis shows the cEFP amplitude during BL. Markers (red = test; blue = sham) below the diagonal represent subjects that during NF reduced cEFP activity relative to BL. 6 out of 7 subjects from the test group could significantly reduce cEFP activity during NF relative to BL compared with only 1 out 6 subjects in the sham group. *p<0.05, **p<0.01, and *n* = 13. For illustration purposes, the cEFP amplitude of the BL for each subject was multiplied by the NF mean. The actual range of the cEFP amplitude during BL was (-0.2)-(0.34).

Success in down-regulating the common EFP signal amplitude was measured by comparing the mean cEFP amplitude during baseline (BL) with the mean amplitude during NF. We hypothesized that only the test group would reduce cEFP amplitude during NF relative to BL. Two-way repeated measures ANOVA revealed a significant interaction between groups (test vs. sham) and conditions (BL vs. NF) (F(1,11) = 11.91, *p*<0.01) (Fig 6a). Planned comparisons revealed that, as expected, whereas the test group significantly down-regulated the cEFP signal during NF relative to BL (F(1,11) = 24.46, *p*<0.01; BL[mean±sd] = 0.01±0.07; NF = -0.84±0.61), the sham group did not (F(1,11) = 0.01, *p*>0.90; BL = 0.01±0.19; NF = 0.03±0.34). No differences were observed between the test and the sham groups during BL (F(1,11) = 0.01, *p*>0.95).

Analyzing NF success at an individual level further indicated that real-time auditory feedback driven by the cEFP amplitude induced learned regulation. Individual success was determined by conducting a single subject GLM (t-test) for each participant between BL and NF (successful subject: *p*(NF < BL) < 0.05). As expected, a significantly higher success rate was observed among the test group than among the sham group (Fisher-exact *p*<0.05; test: 6 out of 7; sham: 1 out of 6) (Fig 6b).

## 4. Discussion

The current study introduced a novel data-driven approach for the construction of an EEG prediction model (EEG finger print; EFP) of localized fMRI-BOLD activity. Extending upon previous work [25] the current study developed a common EFP (cEFP) model of the amygdala that is valid across individuals, thus relieving the necessity of a prior fMRI scan for each subject or session. Implementing the new common model in neurofeedback demonstrated the feasibility of using the cEFP as a neural probe for self-regulation training.

The suggested framework used a one-class ridge regression model to find a time/frequency representation of specific brain activity as measured simultaneously by the fMRI. This data representation approach was used in our previous work [25] (rather than searching the vector of frequency representation [24] or searching a specific HRF for each frequency separately [22,23]) and showed better performance than the current state-of-the-art approaches.

The common model, created using the suggested framework, shows that the theta, alpha, and beta frequency bands contribute to the predicted fMRI BOLD signal modulation in the amygdala. This result is similar to the averaged normalized individual EFPs shown in [25], except the gamma contribution that is absent from the current model. This finding is consistent with prior studies that investigated the EEG spectral power during relaxation and found those frequency bands (occipital-alpha, beta, and theta) to be informative measures for relaxation [44,45,46,47,48]. In addition, during a gradual attempt to relax, results indicate that amygdala activity is correlated with increased power modulation of theta, but decreased modulation of alpha waves [49,50].

The cEFP was constructed using a partial group of sessions belonging to different subjects (*n* = 10) with homogeneous individual EFP characteristics. Therefore, the basic assumption should be that it probably will not provide an accurate predictor of the amygdala for all subjects. To test this claim, the performance of the common EFP was compared to the performance of the previously developed individual EFPs. However, this comparison is unbalanced since the individual EFP model, despite its better performance, requires prior fMRI scanning for each subject and session and is, therefore, not applicable. In addition, the group of new sessions has a biased distribution due to the fact that the best sessions were excluded and used only for the construction of the cEFP model. Therefore, to validate that the cEFP can indeed reliably predict amygdala BOLD activity across different individuals, further research is required, including simultaneous EEG/fMRI with a new sample, not previously used to develop the model. The possibility that the resulting cEFP represents a cortical activity related to the amygdala, and not the amygdala's activity itself should also be the subject of future research. Furthermore, such research should also consider the implications of new findings regarding the reliability of fMRI in measuring amygdala activity [51] and apply the suggested new EPI sequence and other suggested improvements. In addition, the inhomogeneity in the accuracy across different individuals exhibited by the cEFP calls for an ensemble of different one-class models (for different groups of subjects), including the development of an EEG based characterization method to fit different models to different individuals without an fMRI scan.

The feasibility of using the common model as a probe for NF was demonstrated by testing whether participants could learn to down-regulate the signal amplitude. NF success was tested by comparing the cEFP amplitudes of the test and sham groups during NF periods relative to baseline rest periods (BL). The results showed that during NF, subjects that received online auditory feedback of the cEFP learned to down-regulate the signal amplitude. One might argue that the lowering of cEFP amplitude observed in the test group is due to a global effect caused by relaxation and not due to the feedback. In that case, we would expect the same down regulation to be observed in the sham group. Participants of the sham group were blind to their assignment and received an auditory feedback unrelated to their own amygdala activity, indicating successful down regulation. The post training debriefing revealed that none of the participants in the sham group suspected that they were in a control condition. More importantly, they reported the use of relaxation strategies similar to those reported by the test group (mostly self-introspection and guided imagery). We can thus confidently infer that the success in cEFP down regulation was due to the informative real-time feedback of cEFP amplitude modulations. However, whether this learned regulation could facilitate limbic related-processes, as previously demonstrated by fMRI-NF [8,9,52], should be rigorously investigated. Encouraging results from a recent study that conducted fMRI scans before and after amygdala cEFP-NF training suggested that learned down-regulation of the cEFP could result in amygdala related behavioral modifications [53]. By using simultaneous EEG/fMRI with a new sample the above mentioned study also provided reassuring evidence regarding the reliability of the cEFP in predicting the amygdala BOLD activity.

The current framework demonstrated the potential of developing an EEG based model of localized activity in a single region using a single electrode. However, recent evidence suggests that emotional processes are better reflected by the interaction patterns between networks of multiple regions [54], and not only by the activity of a single region. Extending the framework introduced in the current study to multiple electrodes (or network of regions) to model such neural patterns requires further investigation due to the increasing dimension of the feature space. Integrating these models in neurofeedback could have substantial therapeutic and diagnostic potential.
